# Control of *Taenia solium*; A Case for Public and Private Sector Investment

**DOI:** 10.3389/fvets.2019.00176

**Published:** 2019-06-20

**Authors:** Lian F. Thomas, E. Anne J. Cook, Eric M. Fèvre, Jonathan Rushton

**Affiliations:** ^1^Institute of Infection and Global Health, University of Liverpool, Liverpool, United Kingdom; ^2^International Livestock Research Institute, Nairobi, Kenya

**Keywords:** *Taenia solium*, cysticercosis, control, interventions, economics, incentives

## Abstract

The zoonotic helminth *T. solium* is one of the leading causes of acquired epilepsy in endemic countries, resulting in a high burden both in human health and social stigma of affected people (1–3). In 2012 *T. solium* was highlighted as a priority for control in the World Health Assembly resolution 66.12 (4). Despite a call for validated control strategies by 2015 and a “Tool Kit” of control options being available, relatively few examples of successfully implemented and sustainable control programs are available (5–7). A minimal control strategy focusing solely on the porcine host has also been proposed although the cost-effectiveness of such has yet to be explored (8). Although acknowledgment has been made of the need for initiatives to be sustainable, we are yet to see sufficient consideration of the balance between the provision of public and private goods, and the need for engagement of the people and organizations in the pork value chains within *T. solium* control strategies. We utilized a food chain risk analysis model to determine the incremental cost-effectiveness ratio (ICER) in terms of $/infective meal avoided, of combining a pharmaceutical intervention in pigs with strengthened meat hygiene services. The addition of a vaccination and treatment protocol, at an additional 10.3% cost, was illustrated to have the potential to improve the ICER of improving meat inspection by 74.6%. The vaccination and treatment protocol also had the potential to reduce the losses borne by the pork industry of condemned meat by 66%, highlighting the potential to leverage private sector investment in *T. solium* control.

## Introduction

*Taenia solium* is a zoonotic tapeworm which utilizes a porcine intermediate and a human definitive host. It is thought that *T. solium* has been associated with a hominid definitive host pre-dating the advent of *Homo sapiens* (9) and has accompanied modern humans as they colonized the globe (10). Humans acquire a *T. solium* taeniosis infection through consumption of pork containing viable cysticerci and pigs acquire *T. solium* cysticercosis through the ingestion of infective eggs or proglottids excreted in the feces of infected humans (11). The ingestion of infective eggs by humans due to fecal contamination of food or drinking water, or auto-infection from a tapeworm carrier, can lead to an aberrant intermediate infection, cysticercosis, with larval cysts found in muscle, optical or neural tissue. Infection of the central nervous tissue, neurocysticercosis (NCC) is considered to be a major causes of acquired epilepsy in endemic counties (12), leading to significant reductions in quality of life (13) and making *T. solium* the foodborne parasite with the greatest global burden (14).

Improvements in pig production, sanitation and meat hygiene have contributed to the decline in *T. solium* infection pressure in North America and Europe, although pockets of endemnicity exist where a triad of poor sanitation, free-range pigs and lack of food safety governance are found, making it very much a disease of poverty (15). The three major endemic regions are Africa, Asia, and Latin America (16), although there is evidence that the parasite may still have autochthonous transmission within Eastern Europe (17). Even within individual countries in endemic regions the parasite has a varied spatial and temporal distribution depending on local factors influencing the lifecycle (18).

*T. solium* taeniosis/cysticercosis has traditionally been considered one of the neglected zoonotic diseases (19–22) but increased advocacy and a growing body of literature detailing the prevalence and burden of this parasite has led to its incorporation into the 2012 London Declaration on Neglected Topical Diseases (NTDs) (23), The WHO Roadmap “Accelerating work to overcome the global impact of neglected tropical diseases” (24) and the World Health Assembly resolution WHA66.12 (25).

Despite this high-level commitment we have yet to significantly advance the control of the parasite on a large scale. As a community, we have failed to achieve the 2015 goal set by the WHO Roadmap, to have a “validated strategy for control and elimination of *T. solium* taeniosis/cysticercosis available” and are unlikely to meet the 2020 goal for scaled up interventions. A “tool kit” of intervention options are available, each of which has the potential to break the tapeworm lifecycle at different points by focusing on either the human or porcine host (7).

Interventions targeting the human host include improving access to clean water and sanitation, preventative chemotherapy (often in the form of mass drug administration) and wider public health education campaigns (26). Control interventions in the porcine host and associated value chain such as the confinement, anthelmintic treatment or vaccination of pigs can be considered as pre-harvest (26). Post-harvest control covers stringent meat inspection with condemnation of infected meat (27), treatment of meat through freezing (28), gamma-radiation (29), and salt-pickling (30) as well as cooking to over 50°C (30, 31) which also assists in the control of other foodborne pathogens (32–34).

An optimal intervention strategy has not yet been demonstrated in the field and importantly the acceptability and sustainability of these strategies has not been evaluated (6, 35–37), with the cost-effectiveness of control evaluated in only two studies to date (38, 39). A “One Health” approach, with interventions in both the human and non-human animal host has generally been regarded to be necessary for the control of zoonoses such as *T. solium* (40). It has recently been suggested that a “minimal intervention strategy” targeting only the porcine host through vaccination and anthelmintic treatment of pigs between 2 and 7 months of age may be appropriate (8).

The One Health strategy utilizes the TSOL18 vaccine which has demonstrated 100% protection against porcine cysticercosis under field conditions (41) and which is now produced by India Immunologicals Ltd, India as Cysvax® (8). The anthelmintic treatment to be administered is oxfendazole, administered at 30 mg/kg, now available in some African countries as a 10% formulation for pigs (Paranthic) (8). Oxfendazole (30 mg/kg) has also been demonstrated to also have 100% efficacy against the gastrointestinal nematodes *Ascaris suum, Oesophagostomum* spp., *Trichuris suis* and *Metastrongylus* spp., thereby providing additional benefits to productivity for pig farmers (42). A recent trial of this strategy in Nepal demonstrated a significant reduction in porcine cysticercosis, with elimination of infection in those animals assessed by post-mortem (43).

In an integrated control program in Laos PDR, a pig intervention including the TSOL18 vaccine, oxfendazole (30 mg/kg), was combined with *T. solium* and soil transmitted helminth control in humans through the mass administration of Albendazole (44). An economic analysis of this program from a societal perspective has been conducted and the combined approach was judged to be highly cost-effective at 214 USD/DALY averted against the GDP per capita of Laos PDR of 1,793 USD (38).

As yet all studies utilizing porcine pharmaceutical interventions have been provided to farmers free of charge. Whilst acknowledging the barriers, including lack of access to finance or credit to make capital investments of feed purchases (45), to improve the pork value chain in endemic areas, it is important to provide value chain actors with the responsibility and agency to deliver a safe and quality product to market, if sustainable control is to be achieved. We suggest that a cost-sharing model between the private and public sector may be a suitable direction to take for *T. solium* control, based upon the delivery of private (e.g., profit) or public (e.g., food safety) goods through the different control interventions.

We hypothesize that farmers may be incentivized to adopt a control strategy through demonstration of “rewards,” such as increased profitability of the pig production system due to improved husbandry practices or the adoption of judicial use of anthelmintic treatment for gastro-intestinal nematode infections in combination with *T. solium* control. Behavior change may also be encouraged through potential punishments, such as the condemnation of grossly infected meat at inspection.

Despite the low sensitivity of meat inspection for the detection of *T. solium* (46), highly infected carcasses are likely to be observed if qualified personnel are present at slaughter, well trained and sensitized to the importance of preventing consumption of infected meat. If meat inspection is carried out according to regulatory standards, a trader or butcher who presents a pig to slaughter carries the full burden of risk should that carcass be condemned at meat inspection as no compensation is received for condemned animals (47).

Changes in demand, toward uninfected pigs, may induce losses for small-holder pig farmers until they adopt *T. solium* control strategies. It could be hypothesized therefore, that the public expenditure of enforcing meat hygiene regulations may therefore “leverage” investment from the private sector in control measures (48). An example of such would be the purchase of vaccines and anthelmintic treatment for pigs directly by farmers, rather than through publicly funded campaigns.

The current study aimed to explore this hypothesis by determining the incremental cost-effectiveness ratio (ICER) in terms of $/infective meal avoided, of the “minimal intervention strategy” of pharmaceutical intervention in pigs in combination with strengthened meat hygiene services in western Kenya. We utilize a food chain risk analysis modified from Thomas et al. (49) parameterized by data relating to western Kenya although the model parameters may be easily adjusted for use in other settings.

## Materials and Methods

### Study Area

The data used to parameterize the risk model described below was obtained through previously described studies conducted in a mixed crop-livestock farming community in western Kenya, centered around Busia town on the Uganda border (50, 51). Many pig farmers in this area practice extensive forms of pig production, with three systems predominating; full time free range where pigs are left to scavenge for all their food requirements, part-time free-range where pigs scavenge during the dry season, where during planting or growing seasons they may be tethered or confined with supplemental feedstuffs to prevent crop damage, or full time confined systems which may involve tethering or confining pigs in rudimentary structures and providing supplementary feedstuffs (52–54). These systems are similar to those described in other endemic areas (55–60). Within the study site, 16.6% (95% C.I. 13.1–20.5) of homesteads owned pigs, and the majority of farmers sell their pigs to butchers who transport the pigs to rudimentary, but licensed, slaughter premises for slaughter, with a small, but important proportion (4.3%, 95% C.I 2–12%) of pigs undergoing “back-yard” slaughter (52). By law, a meat inspector must inspect each pig, although low staffing levels and poor facilitation in terms of transport, means that many animals are currently slaughtered without inspection.

### Food Chain Risk Analysis

**Figure 1:**
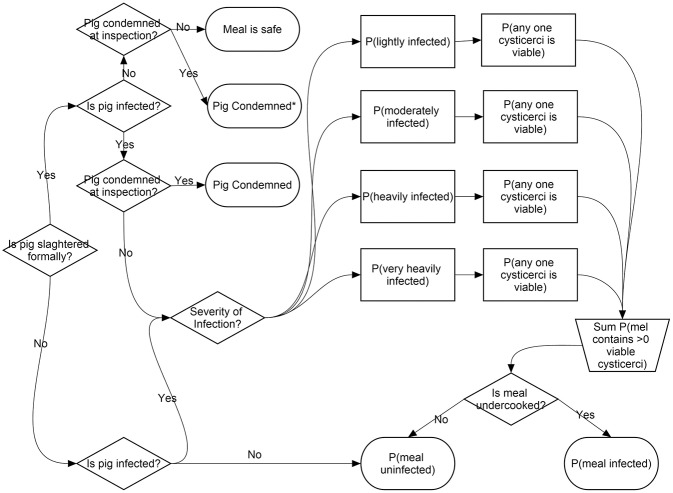
Food chain risk model structure © 2017 Thomas et al. Reproduced from Thomas et al. (49).

A stochastic risk assessment model, built using the @Risk (Palisade, Newfield, NY, USA) add-on for Excel (Microsoft corp., USA) and the initial parameters (P1–P25) are described in detail in Thomas et al. (49). The structure of the model is illustrated in Figure 1 for ease of reference. This model indicates that any one pork meal consumed in western Kenya has a 0.006 (99% Uncertainty Interval (U.I). 0.0002 ± 0.0164) probability of containing at least one viable *T*. *solium* cysticercus at the point of consumption and therefore being potentially infectious to humans (49). We adapted this model to investigate the ICER from a societal perspective of enforcing best practice meat inspection at every registered porcine slaughter facility in Busia county with or without adoption of a regime of Cysvax vaccine and Oxfendazole in pigs at 3 and 6 months of age, adapted from the minimal intervention strategy as recommended by Lightowlers and Donadeu (8).

The assumptions in this adapted model are as follows:

- Pigs are slaughtered between 7 and 12 months of age
- Adoption of vaccination and treatment protocol was assumed to be 75% (70–80%) of farmers fattening pigs for the “formal” value chain (not those destined for “backyard” slaughter)
- Vaccination and treatment at 3 and 6 months (2 doses) is 100% protective (8, 61), but 1% of treatments may fail through user error
- Vaccination and treatment failure will result in infection profiles equivalent to non-treated pigs (i.e., Proportion of light/medium/heavy infections will be equivalent)
- Meat inspectors will be present at every formal slaughter facility and inspect every pig presented
- The proportion of pigs destined for the informal sector from fattening remains at the baseline level and these farmers do not take up the pharmaceutical intervention.
- Pigs slaughtered in the informal sector obtain only 45% of the price of a formally slaughtered pig (62).

The updated model can be found in Supplementary Material S1. The new model parameters are described in Table 1 and the original model parameters are presented in Thomas et al. (49). Ten additional scenarios were added to the original 15 detailed in the original model (49) and are described as follows:

Scenario 1 = Pig is formally slaughtered/treated/lightly infected/not detected at meat inspection
Scenario 2 = Pig is formally slaughtered/treated/lightly infected/detected at meat inspection/condemned
Scenario 3 = Pig is formally slaughtered/treated/moderately infected/not detected at meat inspection
Scenario 4 = Pig is formally slaughtered/treated/moderately infected/detected at meat inspection/condemned
Scenario 5 = Pig is formally slaughtered/treated/heavily infected/not detected at meat inspection
Scenario 6 = Pig is formally slaughtered/treated/heavily infected/detected at meat inspection/condemned
Scenario 7 = Pig is formally slaughtered/treated/very heavily infected/not detected at meat inspection
Scenario 8 = Pig is formally slaughtered/treated/very heavily infected/detected at meat inspection/condemned
Scenario 9 = Pig is formally slaughtered/treated/uninfected/ not detected at meat inspection
Scenario 10 = Pig is formally slaughtered/treated/ uninfected/ detected at meat inspection (false positive)/condemned
Scenario 11 = Pig is formally slaughtered/Not treated/lightly infected/not detected at meat inspection
Scenario 12 = Pig is formally slaughtered/Not treated/lightly infected/detected at meat inspection/condemned
Scenario 13 = Pig is formally slaughtered/Not treated/ moderately infected/not detected at meat inspection
Scenario 14 = Pig is formally slaughtered/Not treated/moderately infected /not detected at lingual palpation/condemned
Scenario 15 = Pig is formally slaughtered/Not treated/ heavily infected/not detected at meat inspection
Scenario 16 = Pig is formally slaughtered/Not treated/heavily infected/detected at meat inspection/condemned
Scenario 17 = Pig is formally slaughtered/Not treated/very heavily infected/not detected at meat inspection
Scenario 18 = Pig is formally slaughtered/Not treated/very heavily infected/detected at meat inspection/condemned
Scenario 19 = Pig is formally slaughtered/Not treated/uninfected/ not detected at meat inspection
Scenario 20 = Pig is formally slaughtered/Not treated/ uninfected/detected at meat inspection (false positive)/condemned
Scenario 21 = Pig is informally slaughtered/not treated/ lightly infected
Scenario 22 = Pig is informally slaughtered/not treated/ moderately infected
Scenario 23 = Pig is informally slaughtered/not treated/ heavily infected
Scenario 24 = Pig is informally slaughtered/not treated/ v. heavily infected
Scenario 25 = Pig is informally slaughtered/not treated/ uninfected

**Table 1:** New model parameters. Parameters P1–P25 as per original model (49).

**Parameter**	**Description**	**Source**	**Probability**	**Distribution**
P26	Probability infected pig is detected and condemned at inspection	Sensitivity and Specificity of inspection (46)	0.387 (97.5% C.I 0.22–0.58) Sensitivity	BetaPert (0.1, 0.387, 0.9)
P27	Probability uninfected pig passes inspection		1.0 (97.5% C.I. 0.9-1.0)	BetaPert (0.9, 1.0, 1.0)
P28	Probability pig slaughtered formally has undergone vaccination and treatment protocol	Assumption	0.75	Uniform (0.7-0.8)
P29	Probability pig is not infective after vaccination and treatment protocol	100% effective (8, 61) potential for 1% treatment error	1.0 (0.99–1.0)	BetaPert (0.99, 1.0, 1.0)
P30	Value of carcass at slaughter	Mean dressed-weight 22.5kg (63) Pork price/kg Per. Comms. M.K Murungi, 2018 $3.2	Calculated as dressed-weight × pork price (Static)	
P31	Average daily weight gain	(64)	110 g/day (80–140 g)	BetaPert (80, 110, 130)
P32	Pig live-weight at 3mths		Calculated 8 kg weaned weight + [(P30^*^30)^*^2]	
P33	Pig live-weight at 6mths		Calculated as P32 + [(P30^*^30)^*^3]	
P34	Cost of 1 dose Cysvax (IIL India)	Per. Comms. M. Lightowlers 2018	$0.5	Static
P35	Cost of oxfendazole treatment/kg (Paranthic from MCI Morocco)		$0.00038	Static
P36	Cost of vaccination and treatment protocol per pig	2 doses of Cysvax and 2 treatments with oxfendazole 30 mg/kg (8)	Calculated as (P34 ×2) + (P35^*^32) + (P35^*^P33)	
P37	Number of meat inspectors required to fill deficit in Busia county	Dr Ogendo, County Director of Veterinary Services 2018	24	Static
P38	Global cost per meat inspector (salary, motorbike, ancillary costs)		$164,100	RiskPert(6400,7000,7900)4
P39	Meat Inspection costs		Calculated as P38^*^P37	

The probability of each scenario described is calculated as follows:

$$\begin{array}{l} \text{P}(\text{scenario x})=(\text{P}{\left(\text{formal}/\text{informal slaughter}\right)}^{*}\text{P}(\text{treated})\\ \;*(\text{P}{\left(\text{Infected}/\text{uninfected}\right)}^{*}\text{P}(\text{severity of infection})\\ \;*\text{P}(\text{detected}/\text{undetected at meat inspection})) \end{array}$$

And the probability of any one meal being potentially infective at consumption expressed as:

$$\begin{array}{l} \text{ P}(\text{anyoneporkmealisinfectiveatconsumption})\\ =((\text{P}{\left(\text{pork meal contains a cyst| Situation1}\right)}^{*}\text{P}(\text{Situation1})\\ +\text{P}{\left(\text{pork meal contains a cyst|Situation2}\right)}^{*}\text{P}(\text{Situation2})\\ +\text{P}{\left(\text{pork meal contains a cyst|Situation3}\right)}^{*}\text{P}(\text{Situation3})......\\ +\text{P}{\left(\text{pork meal contains a cyst|Situation36}\right)}^{*}\text{P}(\text{Situation36}))\\ \;{\;}^{*}\text{P}(\text{anyonecystisviablepriortocooking}){)}^{*}\text{P}(\text{Meal undercooked}) \end{array}$$

Only partial costs to the pig industry were considered in this analysis, including; the vaccination and treatment protocol, and losses due to carcass condemnation (pork price/kg × carcass weight), feeding and transport of pigs were not included. Costs to the county government considered are the additional cost of staffing all pork slaughter facilities with a qualified meat inspector. The income from meat inspection fees ($1.4 per pig) were not included as these are currently paid for every pig irrespective of the presence of a meat inspector. The interventions are compared through their incremental cost-effectiveness analysis (ICER), calculated according to the equation (65)

$$\begin{array}{l} ICER=\frac{\left(\text{Cost of strategy }-\text{ Cost current strategy}\right)}{\left(\text{Effectiveness of strategy }-\text{ Effectiveness current strategy}\right)} \end{array}$$

Where: Costs are in US$ at 2017 values

A sensitivity analysis was conducted according to the method described previously (49) to determine the most influential parameters on the ICER output.

## Results

The models converged after 48,900 iterations, a summary of the results can be found in Table 2. All model inputs and outputs can be found in Supplementary Table 1 (improved meat inspection only) and Supplementary Table 2 (Meat inspection and treatment protocol).

**Table 2:** Model outputs.

	**Baseline (no pigs inspected) (49)**	**All pigs presented for slaughter at registered facilities (“formal slaughter”) are inspected**	**Farmers utilize treatment protocol for those pigs entering the formal system**
Estimated risk of infection from any one pork meal consumed	0.006 (99% Uncertainty Interval (U.I). 0.0002–0.0164)	0.0036 (99% U.I 0.00009–0.0118)	0.0012 (99% UI. 0.00003–0.0041)
Number of infective events/year in Busia county	22,282^*^ (99% U.I. 622 ± 64,134)	14,709 (99% U.I. 368-52,209)	5,121 (99% U.I. 118-18,087)
Potentially infective events avoided from baseline	N/A	7,500 (one-sided 99% U.I 0-21,912)	17,161 (99% U.I. 504-46,047)
Losses through condemnation of carcasses	$10,665^*^ (99% U.I. 652-32,200)	$196,078 (99% UI 63,067–395,189)	$67,143 (99% U.I 19.936–138,946)
Total treatment costs	N/A	N/A	$17,363 (99% U.I. 14,828–19,825)
Cost to county government for meat inspection services	$112,817 (99% U.I. 103,712–123,658)	$282,043 (99% U.I. $259,279–$309,146)	$282,043 (99% U.I. $259,279–$309,146)
Condemnation losses avoided through treatment	N/A	N/A	$178,724 (99% U.I. 48,239–375,364)
Incremental cost of intervention from baseline	N/A	$354,730 (99% U.I. 219,694–$555,247)	$239, 102 (99% U.I. $178,999–$352,102)
ICER ($/infective event avoided)	N/A	$59 (99% U.I $15–$402)	$15 (99% U.I.$ 9.81–$33.75)

* *Fixed as a static baseline for comparison with intervention models*.

The model suggests that within the context of an improved meat hygiene service, addition of a vaccination and treatment protocol in pigs has the potential to improve the cost-effectiveness of the intervention by 74.6% from $59 (99% U.I $15–$402) to $15 (99% U.I.$ 9.81–$33.75) per infective meal avoided. For farmers, the cost-benefit ratio for adopting the vaccination and treatment protocol is 10.29, due to the resultant reduction in condemnation losses, without considering the potential additional benefits from increase in weight-gain though treatment of gastro-intestinal nematode infections.

Spearmans rank order coefficients (ρ) indicated that the five most influential inputs on the ICER were; the probability of any one cysticercus being viable (ρ = 0.76), the probability that an uninfected pig is correctly passed at meat inspection (ρ = −0.26), the probability that an untreated pig is infected (ρ = 0.22), the probability of a pig being treated (ρ = −0.18) and the mean number of cysts in a heavily infected pig (ρ = 0.17). These five parameters were included in an advanced sensitivity analysis. If all other parameters are fixed, the probability of any one cyst being viable has the largest influence over the ICER, with the mean at 1% of the input value being $11.06 and at 99% of the input value being $22.4.

## Discussion

The analysis indicates that from a societal perspective, implementing a vaccination and treatment protocol in pigs has the potential to enhance the incremental cost-effectiveness ratio ($/potential infective event avoided) of a *T. solium* control intervention based on enforcing meat inspection regulations. It also indicates the potential for public sector investment, in this case in the meat hygiene inspectorate, to leverage private sector investment, e.g., in a vaccination and treatment protocol for pigs, to “insure” the private sector against potential losses due to regulatory standards.

Within the immediate aftermath of tightening meat hygiene regulations it is expected that food producers will incur a degree of financial loss as they adapt to the new regulatory environment (66). Increased costs may relate to carcass or partial carcass condemnation, or from the increased time required for stringent meat inspection to occur. However, it would also be expected that over time these losses would reduce and stabilize as the market adapts to the new environment, with pork traders and butchers seeking pigs from “improved” producers, or pre-screening pigs for infection prior to purchase, in order to reduce their risk. Screening of pigs by pork traders using lingual palpation has already been reported in Tanzania (67) and Zambia (62) and traders in Kenya have expressed an interest in “insurance” against condemnation (47).

Providing small-scale farmers with the responsibility and agency to bring a safe product to market is an important aspect of improving and growing a viable pig industry in *T. solium* endemic areas. How farmers address the problem of *T. solium*, alongside other animal health and husbandry issues they may be facing, is essentially an individual decision and the solutions chosen must be relevant to the context in which they are operating. Encouraging farmers to invest in *T. solium* control interventions may require a “carrot and stick” approach including enforcement of meat hygiene regulations and promotion of the potential profits afforded by producing “safe” pork.

A combination of rewards and punishments, “carrots and sticks,” have been demonstrated to have a stronger effect on eliciting “correct” behavior, than either alone (68). In terms of rewards to the farmer for adopting such pharmaceuticals there are two potential ways in which revenue may be enhanced. The use of oxfendazole also has the potential to improve the profitability of pig farming through the treatment of the gastro-intestinal nematode infections which are prevalent in many small-holder pig systems. An overall gastro-intestinal nematode prevalence of 91% was detected in small-holder pigs in Uganda (69) and of 84.2% in western Kenya (70). Treatment of these infections should lead to improvement in the feed conversion efficiency of these pigs, leading to increased daily weight gain, as has been demonstrated in cattle (71).

Rewards may also come in the form of a price premium, or enhanced market access, for a high quality product, the goal of many private food standards. Willingness-to-pay for pork perceived to be “safe” has been previously demonstrated in China (72), but the ability to pay a “safe pork” premium assumes a level of disposable income which will allow a degree of inelasticity of demand.

In China, where pork is a traditional component of the diet, the price elasticity of pork has been shown to be low (73). In sub-Saharan Africa pork is not a traditional food, though as populations urbanize and incomes rise there is a rapid increase in the volume of pork consumed in the region (74). In Kenya the price elasticity of pork across rural and urban households was also found to be inelastic at 0.96, although closer to the threshold for a “luxury good” than beef, chicken, and goat (75).

Consumption of pork in much of the region is still predominately the domain of those in the upper income brackets. In Kigali, Rwanda for instance pork has been referred to as “Benz” (as in Mercedes Benz) designating it as a high-status product (76), in Uganda the consumption of pork has been shown to be significantly higher among families of higher socio-economic status (77). Within this demographic there may be an ability to pay such a “safe pork premium,” but willingness-to-pay for safe food is not only a product of consumer incomes, but of education, risk perception, cultural food preferences, and access to substitute foodstuffs or food suppliers (72, 78).

Although the model presented here is of course only an approximation of reality, with many assumptions incorporated, it illustrates how providing pig farmers with access to pharmaceutical products such as the Cysvax vaccine and oxfendazole, could substantially reduce exposure of consumers to a dangerous zoonotic infection as well as reduce potential losses to the pork industry from the condemnation of pig carcasses, or through the sale of infected pork through the “informal” sector, assuming that these pigs obtain only 45% of the market value (62).

Field trials have indicated the efficacy of the vaccination and treatment protocol to reduce the prevalence of porcine cysticercosis (43, 61, 79). Studies are now needed to establish farmers' willingness-to-pay for these pharmaceutical products and the likelihood of uptake in the context of different regulatory frameworks. In order to allow smallholder farmers in endemic areas to adopt vaccination and treatment protocols, products must be available through local suppliers of agro-veterinary products, they must be appropriately packaged in appropriate dosages for smallholder famers who own 1–5 pigs and sufficient extension services should be provided to raise awareness of the products.

Protecting the food chain through meat inspection requires that countries formulate and enact appropriate meat hygiene legislation and also that sufficient staff are deployed and facilitated including across potentially inaccessible rural areas. Within the analysis presented here, the total cost of meat inspection services has been allocated to *T. solium* control, although these services provide goods far beyond this goal. Meat inspection, including *ante mortem* inspection of the animals arriving for slaughter, provides wider benefits than purely cysticercosis control. By removing diseased animals from the food chain, inspection aims to both reduce zoonotic disease exposure to people and to assist in the detection and control of some livestock diseases thereby providing public goods, which cannot be appropriated by any one individual, to both consumers and the livestock sectors, respectively (80). Meat inspectors, or official veterinarians at meat processing facilities, also have a role in ensuring facility hygiene, a role which provides possibly the most important control on microbial contamination of meat products (81).

Regulatory impact assessments and cost-benefit analysis of meat hygiene regulations would be highly useful for policy makers within endemic counties to enable more efficient allocation of resources within already stretched public budgets. Meat inspectors in Kenya are also trained animal health assistants and their role also incorporates aspects of farm extension and surveillance activities. Ongoing work in western Kenya on the surveillance of zoonotic diseases will enable us to begin quantifying the cost-effectiveness of deploying these professional resources across a range of different surveillance and extension activities. Providing economic data will allow countries to prioritize interventions for the NTDs as they move into the next phase of the roadmap to 2030 (82).

## Conclusions

Through the use of a stochastic risk model, we have demonstrated how within the context of enforced meat hygiene legislation, adoption of a porcine vaccination and treatment protocol by farmers may provide a quantifiable economic benefit to the pig industry through a reduction in losses through condemnation. A porcine formulation of oxfendazole (as Paranthic 10%) and TSOL18 (Cysvax) are now in commercial production and licensing is underway in several sub-Saharan African countries, including Kenya. Programmes are now urgently needed to provide access to these products to those who require them, stimulate demand and monitor the uptake and cost-effectiveness of these products if we are to be successful in the global goal to control this important zoonotic parasite.

## Author Contributions

LT conceived and produced a first draft of the manuscript. EC, EF, and JR were involved in the development of the ideas presented in the manuscript and assisted in the writing. All authors read and approved the final draft of the manuscript.

### Conflict of Interest Statement

The authors declare that the research was conducted in the absence of any commercial or financial relationships that could be construed as a potential conflict of interest.

## Supplementary Material

Supplementary Material S1 Adapted food chain risk model. Available online at: https://figshare.com/s/42e6a65deaafc72e1cb6.

Supplementary Table 1 Model inputs and outputs improved meat inspection only.

Supplementary Table 2 Model inputs and outputs improved meat inspection and pharmacological inputs.
